# γ-Aminobutyric acid is involved in overlapping pathways against chilling injury by modulating glutamate decarboxylase and defense responses in papaya fruit

**DOI:** 10.3389/fpls.2023.1233477

**Published:** 2023-11-16

**Authors:** Ghulam Khaliq, Sajid Ali, Shaghef Ejaz, Gholamreza Abdi, Yahya Faqir, Jiahua Ma, Mohammed Wasim Siddiqui, Asgar Ali

**Affiliations:** ^1^ Department of Horticulture, Faculty of Agriculture, Lasbela University of Agriculture, Water and Marine Sciences, Uthal, Pakistan; ^2^ Department of Horticulture, Faculty of Agricultural Sciences and Technology, Bahauddin Zakariya University, Multan, Pakistan; ^3^ Department of Biotechnology, Persian Gulf Research Institute, Persian Gulf University, Bushehr, Iran; ^4^ Engineering Research Center for Biomass Resource Utilization and Modification of Sichuan Province, Southwest University of Science and Technology, Mianyang, China; ^5^ Department of Food Science and Post-Harvest Technology, Bihar Agricultural University, Sabour, India; ^6^ Centre of Excellence for Postharvest Biotechnology (CEPB), School of Biosciences, University of Nottingham Malaysia, Semenyih, Malaysia

**Keywords:** papaya, GABA, chilling injury, abiotic stress, proline, GAD

## Abstract

The effect of γ-aminobutyric acid (GABA) treatment at two concentrations (1 mM or 5 mM) on papaya fruit stored at 4°C and 80%–90% relative humidity for 5 weeks was investigated. The application of GABA at 5 mM apparently inhibited chilling injury, internal browning, electrolyte leakage, malondialdehyde (MDA), hydrogen peroxide (H_2_O_2_), polyphenol oxidase (PPO), phospholipase D (PLD), and lipoxygenase (LOX) activities of papaya fruit. Fruit treated with 5 mM GABA enhanced the activities of ascorbate peroxidase (APX), catalase (CAT), glutathione reductase (GR), superoxide dismutase (SOD), glutamate decarboxylase (GAD), and phenylalanine ammonia-lyase (PAL). In addition, GABA treatment significantly displayed higher levels of proline, endogenous GABA accumulation, phenolic contents, and total antioxidant activity than the nontreated papaya. The results suggested that GABA treatment may be a useful approach to improving the chilling tolerance of papaya fruit by reducing oxidative stress and enhancing the defense system.

## 1 Introduction

Papaya (*Carica papaya* L.) fruit is cultivated and consumed worldwide in tropical and subtropical regions. It contains vitamins A and C, thiamine, riboflavin, polyphenolic compounds, and carotenoids (Parven et al., 2020). The main carotenoids in papaya are β-carotene, β-cryptoxanthin, and lycopene. However, papaya is a typical climacteric fruit, and its ripening is accompanied by a high respiration rate and ethylene production that leads to rapid pulp softening and sudden biochemical changes (Gao et al., 2020). All these changes affect the quality and shelf life of papaya fruit during storage and transportation. These postharvest losses adversely affect the grower’s income and the papaya fruit industry. Therefore, low-temperature storage is one of the best practices to maintain quality and slow down the biochemical and physiological processes of papaya fruit. Unfortunately, papaya fruit is very vulnerable to chilling temperatures when kept below 10°C (Wu et al., 2019). Papaya fruit shows chilling injury symptoms such as water soaking, surface pitting, shriveling of peel, internal browning, flesh mealiness, and poor aroma and flavor, which leads to a short storage life and lower fruit quality (Pan et al., 2019).

Two main hypotheses were suggested to elucidate the effect of low temperatures on chilling-sensitive plants. The first assumption states that the cell membrane is the main site for chilling injury development (Liang et al., 2020). The biophysical structure of the cell membrane changes under low-temperature stress, which may cause decreased fluidity, loss of membrane function, and deactivation of membrane-bound enzymes, ultimately destroying the membrane (Wolfe, 1978). Lipid peroxidation induces the solidification of saturated fatty acids and increases the ratio of sterol/phospholipid, enhancing membrane fluidity (Marangoni et al., 1996). This reveals that changes in the physical properties of cell membranes are temperature-dependent, and during low-temperature stress, these induced changes can be reversed before irreparable damage to the cell membrane occurs. The second assumption describes that during chilling stress, plants have been shown to produce higher levels of reactive oxygen species (ROS) that comprise hydrogen peroxide, hydroxyl radical, and superoxide anion (Valenzuela et al., 2017). ROS are the main cause of lipid peroxidation, and this leads to cellular disorders and the manifestation of chilling injury symptoms (Liang et al., 2020). Plants have two defense systems to scavenge ROS. The first is antioxidant compounds such as ascorbate, glutathione, vitamin A, phenolics, and anthocyanins. The second system is composed of antioxidant enzymes like APX, CAT, GR, and SOD. In response to oxidative stress, plants raise their enzymatic and nonenzymatic antioxidant defense systems to moderate the severe effects of stress caused by ROS (Li et al., 2019).

A lot of postharvest methods have been developed to maintain the quality and regulate chilling injury of various fruits, including edible coatings (Mendy et al., 2019), low- and high-temperature conditioning (Jin et al., 2014), modified atmosphere (Liang et al., 2020), and elicitors, including exogenous methyl jasmonate (Cao et al., 2010; Chen et al., 2021), melatonin (Mirshekari et al., 2020), nitric oxide (Wang et al., 2016), oxalic acid (Li et al., 2014), and glycine betaine (Luo et al., 2022). All these techniques have played an effective role in reducing chilling injury and maintaining the quality of fruits, but the efficiency of these methods depends on the cultivar, preharvest factors, storage temperature, and duration of storage (Zou et al., 2014). In addition, these methods have limitations due to complicated operations, high energy consumption, chemical residues, and high investment. Modified atmosphere storage is an efficient technique to reduce chilling injury in fruits; however, it changes the gas composition, resulting in undesirable effects like off-flavor and anaerobic respiration. Therefore, it is necessary to discover simple, inexpensive, safe, and efficient approaches to mitigate the chilling injury to papaya fruit. The activation of resistance mechanisms through physical or chemical treatment is attracting great attention for maintaining fruit quality and reducing oxidative damage (Ding et al., 2019).

γ-Aminobutyric acid is a non-protein amino acid and is involved in many physiological processes as a signaling molecule. GABA is a natural compound that regulates oxidative stress responses such as drought, heat, ultraviolet irradiation, and chilling (Ramos-Ruiz et al., 2019). GABA is considered to be involved in the activation of defense mechanisms, induction of nitrate transport, pollen tube growth, cell elongation, anti-chilling protection, and advancement of plant growth and development (Rastegar et al., 2020). GABA is associated with numerous physiological processes like the regulation of cytosolic pH, carbon flux into the tricarboxylic acid cycle, redox status, osmoregulation, and energy production (Ramos-Ruiz et al., 2019). The evidence revealed that GABA treatment inhibited the synthesis of saturated fatty acids and the accumulation of ROS and triggered the antioxidant defense system (Aghdam et al., 2016).

GABA is synthesized through a GABA shunt pathway, which consists of the three enzymes GABA transaminase, glutamate decarboxylase (GAD), and succinic semialdehyde dehydrogenase. GAD activity is primarily responsible for the accumulation of GABA, which contributed to the improvement of chilling tolerance in fruits stored in cold storage (Shang et al., 2011). Through the increase of GAD activity and the regulation of GABA-T activity, exogenous GABA treatment may influence the GABA shunt pathway during cold storage, resulting in GABA accumulation that may help as an adaptive defense mechanism against chilling stress. In addition to participating in the regulation of osmotic balance and enhancing stress tolerance, proline accumulates in plants during chilling stress (Wang et al., 2014). Proline plays a key role in the prevention of chilling injury because higher proline concentrations under chilling stress lead to higher chilling tolerance. Due to increased GAD, pyrroline-5-carboxylate synthetase (P5CS), and ornithine δ-aminotransferase (OAT) activity during storage, proline contents increased in peach fruit during storage. A reduced proline dehydrogenase (PDH) activity is correlated with an increase in proline contents because PDH is the rate-limiting mitochondrial enzyme that catalyzes proline into glutamic acid (Shang et al., 2011). Therefore, treated fruits with higher proline levels and increased GAD activity respond more actively to chilling stress, and their accumulation increases the ability of fruits to improve chilling tolerance during storage.

GABA treatments increased resistance to chilling stress in several fruits, such as peaches (Shang et al., 2011; Yang et al., 2011), bananas (Wang et al., 2014), citrus (Sheng et al., 2017), and pears (Li et al., 2019). It was reported that GABA treatment induced the accumulation of proline contents, activated antioxidant enzymes, and maintained cellular membrane integrity (Malekzadeh et al., 2017). The exogenous application of GABA has similar effects as the endogenous molecule, enabling plants to cope with stress conditions. However, no report is available for the GABA treatment of the chilling injury to papaya fruit. For the first time, the present work provides an innovative insight into the role of exogenous GABA treatment in the regulation of the GABA-shunt pathway by activating proline metabolism and GAD activity in papaya fruit. Therefore, the objectives of this study were to investigate the crucial role of GABA in mediating the resistance mechanism, oxidative stress tolerance, and antioxidant defense system of papaya fruit during low-temperature storage.

## 2 Materials and methods

### 2.1 Fruit materials and treatment

Papaya fruit was harvested at the physiologically mature stage from an orchard located in Uthal, Balochistan, Pakistan. After harvest, all fruit were shifted to the laboratory within 1 h. Harvested fruits with uniform size, maturity, color, smooth surface, no disease symptoms or cracks, and free from mechanical damage were selected for the experiment. Based on our preliminary study, GABA was tested at different concentrations; however, 1 mM to 5 mM GABA treatments were found safe and had no adverse effect on papaya. The fruits were divided into three groups of 90 fruits each, and each replicate contained 30 fruits. The first and second groups were immersed in 1 mM and 5 mM GABA solution, respectively, for 5 min. The third group was dipped in distilled water for 5 min and served as a control. Each treatment was replicated three times. All the fruits were then air dried for 1 h and stored at 4°C (80%–90% RH) for 5 weeks. The biochemical observations were measured at 0, 1, 2, 3, 4, and 5 weeks of cold storage. The chilling injury index, internal browning, and electrolyte leakage were determined after 4 h of the sample transferred from cold storage. The flesh tissues of the fruit sample were taken and stored at −80°C (Ultra Low-Temperature Freeze, DW-HL528S, China) for subsequent analysis.

### 2.2 Measurement of chilling injury and internal brown index

The chilling injury (CI) index was assessed after 4 h of the sample being transferred from cold storage. The chilling injury symptoms were assessed visually on the fruit surface. The chilling injury was measured on each fruit using a five-point scale from 0 to 4: 0 = no symptoms; 1 = trace injury (1%−20%); 2 = slight injury (20%−40%); 3 = moderate injury (40%−60%); and 4 = severe injury (>60%). The chilling injury was estimated using the following formula: CI index = Σ[(CI ranking) × (number of fruit at CI ranking)]/(total number of fruit × highest CI ranking) × 100.

The internal browning (IB) index was measured in the mesocarp area based on the total browning symptoms using the following subjective scale: 0 = no browning; 1 = browning area ranging from 1% to 20%; 2 = browning area ranging from 20% to 40%; 3 = browning area ranging from 40% to 60%; 4 = browning area >60%. The internal browning index was assessed using the following formula: IB index = Σ[(IB ranking) × (number of fruit at IB ranking)]/(total number of fruit × highest IB ranking) × 100.

### 2.3 Electrolyte leakage

Electrolyte leakage (EL) was determined according to the method of Khaliq et al. (2016) with slight modifications. Using a cork borer with a 10-mm diameter, 15 discs (4 mm thick) of papaya flesh were excised from the middle part of the fruit. The discs were put in 25 mL of deionized water and shaken constantly for 30 min. Electrolyte leakage (*L*_0_) in the solution was assessed using a conductivity meter (BANTE, DDS 12DW, USA). The discs were then placed in a boiling water bath for 15 min. After cooling, the electrolyte leakage (*L*_1_) was remeasured. The results were then estimated using the following formula: EL (%) = (*L*_0_/*L*_1_) × 100.

### 2.4 Malondialdehyde content

The MDA content was measured according to the method described by Dhindsa et al. (1981). Using a refrigerated centrifuge D3024R, USA, one gram of sample tissue was homogenized with 3 mL of 5% (w/v) trichloroacetic acid before being centrifuged at 12,000×*g* for 20 min at 4°C. Afterwards, 2.5 mL of 0.5% thiobarbituric acid was mixed with the supernatant (1.5 mL). The reaction solution was heated in boiling water for 30 min and then centrifuged at 10,000×*g* for 10 min. The absorbance was measured at 532 nm, 600 nm, and 450 nm using a UV/Vis spectrophotometer (T80, UK). The results were expressed as micromoles per kilogram of fresh weight (FW).

### 2.5 Determination of hydrogen peroxide

Hydrogen peroxide was determined following the method of Patterson et al. (1984). Two grams of sample tissue was homogenized with 5 mL of ice-cold acetone and centrifuged at 10,000×*g* for 15 min at 4°C. One milliliter of the supernatant was mixed with 0.2 mL of concentrated ammonia and 0.1 mL of 5% titanium sulfate. The peroxide–titanium complex was precipitated. The precipitate was mixed with 4 mL of 2 M sulfuric acid and then centrifuged at 3,000×*g for* 10* min*. The absorbance was measured at 415 nm. A standard curve was constructed with H_2_O_2 at_ concentrations ranging from 10 μM to 100 μM, and the results were expressed as millimoles per kilogram of FW.

### 2.6 Endogenous GABA content and GAD activity

The GABA content was measured following the method of Hu et al. (2015). One gram of fruit sample was mixed with 3 mL of 0.05 M lanthanum chloride, and the mixture was centrifuged for 5 min at 12,000×*g* at 4°C. Afterward, the supernatant was added with 200 μL of 2 M potassium hydroxide and again centrifuged for 5 min at 12,000×*g* at 4°C. Consequently, 400 μL of supernatant was mixed with 600 μL of 0.05 M phosphate buffer (pH 10), 5% NaOCl, and 200 µL of 6% concentrated phenol in boiling water for 10 min. Subsequently, 100 μL of 60% alcohol was added to the reaction mixture. The absorbance at 645 nm was monitored using a spectrophotometer. The GABA content was quantified with a standard curve constructed using known amounts of GABA and expressed as milligrams per kilogram of FW.

For GAD enzyme extraction, 2 g of sample tissue was added to 5 mL of extraction Tris-HCl (0.1 M, pH 9.1) buffer containing 10% glycerol, 0.5 mM of pyridoxal phosphate, 1 mM of phenylmethylsulfonyl fluoride, 1 mM of dithiothreitol, and 5 mM of ethylene diamine tetraacetic acid. The mixture was centrifuged at 12,000×*g* for 20 min at 4°C, and the resulting supernatant was used for GAD determination. The activity of the GAD enzyme was assayed according to the method of Deewatthanawong et al. (2010). The enzyme assay contained 0.1 M of potassium phosphate buffer (pH 5.8), 40 μM of pyridoxal phosphate, and 3 mM of l-glutamic acid. The reaction was ended by adding 0.1 mL of 0.5 M hydrochloric acid. GAD activity was expressed as units per gram per hour of fresh weight.

### 2.7 Proline content

The proline content was determined following the method of Shang et al. (2011). In brief, 1 g of fruit sample was homogenized with 5 mL of 3% (v/v) sulfosalicylic acid and centrifuged at 12,000×*g* for 10 min at 4°C. Two milliliters of the supernatant was added to 3 mL of ninhydrin reagent and 2 mL of glacial acetic acid and boiled at 100°C for 1 h. After cooling, 4 mL of toluene was added to the reaction mixture. The absorbance was recorded at 520 nm. A standard curve was constructed using a known concentration of proline, and the results were expressed as milligrams per kilogram of FW.

### 2.8 Extraction and assays of antioxidant enzyme

For enzyme extraction, 5 g of flesh samples was homogenized in 10 mL of ice-cold extraction sodium phosphate buffer (100 mM, pH 7.5) containing 2% PVPP and 1 mM ethylene diamine tetraacetic acid. The mixture was centrifuged at 12,000×*g* for 20 min at 4°C, and the resulting supernatant was used for the enzyme assay.

CAT activity was measured according to the method of Zhang et al. (2013). CAT activity was expressed as units per gram per minute. GR activity was analyzed following the method of Ding et al. (2007). GR activity was expressed as units per gram per minute. APX activity was determined following the method of Zhang et al. (2013). APX activity was expressed as units per gram per minute. Superoxide dismutase (SOD) activity was analyzed according to the method of Zhang et al. (2013). SOD activity was expressed as units per gram per hour.

### 2.9 PAL and PPO activity

PAL activity was assayed following the method of Nguyen et al. (2003). Five grams of sample tissue from the pulp was homogenized in 10 mL of sodium borate buffer (100 mM, pH 8.8) containing 1% PVPP and 5 mM β-mercaptoethanol. The homogenate was centrifuged at 13,000×*g* for 20 min at 4°C, and the resulting supernatants were collected for the enzyme assay. PAL activity was expressed as units per gram per minute. PPO activity was assayed following the protocol of Nguyen et al. (2003). Five-gram sample tissue was mixed with 10 mL of phosphate buffer (100 mM, pH 7.8) and 1% PVPP. The mixture was then centrifuged at 13,000×*g* for 20 min at 4°C. The supernatant was used for the PPO enzyme assay. PPO activity was expressed as units per gram per minute.

### 2.10 PLD and LOX enzyme activity

The activities of PLD and LOX enzymes were measured in accordance with the procedure outlined by Aghdam et al. (2016). One unit of PLD was defined as the quantity of enzyme required to catalyze the synthesis of 1 nmol d-nitrophenol h^−1^. One unit of LOX was defined as the amount of enzyme that induced an increase in absorption of 0.01 min^−1^ at 234 nm.

### 2.11 Measurement of total phenolics and antioxidant activity

Total phenolic contents were estimated following the Folin-Ciocalteu reagent method as described by Farina et al. (2020). The content of total phenols was expressed in terms of grams of gallic acid equivalents per kilogram of sample fresh weight, using gallic acid as a standard. The free radical 2,2-diphenyl-1-picrylhydrazyl (DPPH) scavenging method was assessed to quantify the total antioxidant activity according to Rastegar et al. (2020). DPPH scavenging activity was expressed as a percent.

### 2.12 Statistical analysis

A two-level factorial completely randomized design (CRD) with three replications was used in this study. Using SAS software, data on the effects of GABA treatment on the biochemical and physiological characteristics of papaya fruit were analyzed. The Fisher’s least significant differences (LSD) test was used for *post-hoc* analysis. All model parameters were tested at *p* < 0.05 for significance level.

## 3 Results

### 3.1 Chilling injury and internal browning

**Figure 1:**
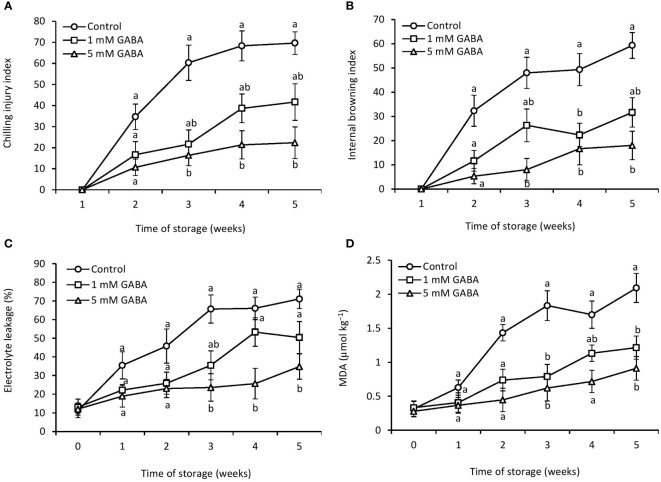
Chilling injury **(A)**, internal browning **(B)**, electrolyte leakage **(C)**, and MDA **(D)** of papaya fruit treated with GABA during storage at 4°C for 5 weeks. Vertical bars represent the standard error of the means for three replicates. Means with different letters show significant differences.

**Figure 2:**
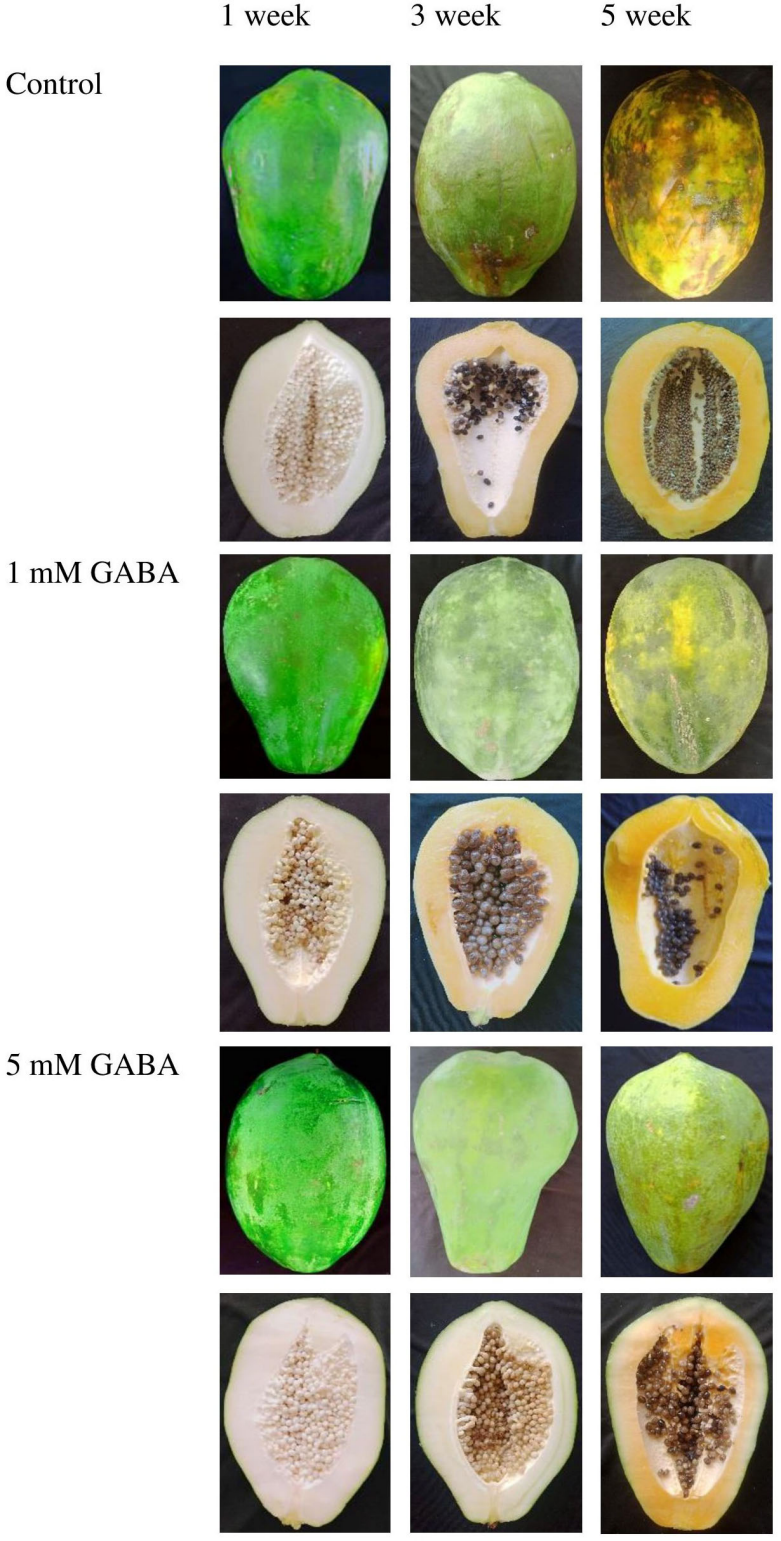
Peel and pulp appearance of papaya fruit treated with GABA during storage at 4°C for 1, 3, and 5 weeks.

The chilling injury index of papaya increased after 2 weeks of storage in both GABA-treated and control fruit. However, this increasing trend was postponed when fruit was subjected to GABA treatment. The chilling injury index was significantly reduced in papaya fruit treated with 1 mM or 5 mM GABA compared to the control (**Figure 1A**). The most effective result was observed in fruit at a concentration of 5 mM GABA treatment. The internal browning appeared after 2 weeks of storage, and afterward, it continuously increased in both control and GABA-treated fruit over the rest of the storage period (**Figure 1B**). The exogenous application of GABA treatment efficiently inhibited the development of internal browning, which is a typical chilling injury symptom in papaya fruit. GABA treatments not only resulted in a lower chilling injury from 2 to 5 weeks but also inhibited the increase in internal browning during the whole storage period compared to the control fruit (**Figure 2**).

### 3.2 Electrolyte leakage

Cell membrane integrity can be measured by electrolyte leakage. Electrolyte leakage increased over the entire storage period, irrespective of the GABA treatments (**Figure 1C**). After 5 weeks of storage, electrolyte leakage in papaya fruit treated with 1 mM or 5 mM GABA was 29% and 52% lower, respectively, than the control fruit.

### 3.2 MDA content

The increased production of MDA reflects oxidative deterioration and damage to cell membranes. The amount of MDA increased with increasing the storage period. However, the exogenous application of GABA led to a reduction of lipid peroxidation (**Figure 1D**). The lowest level of MDA was observed in fruit treated with 5 mM GABA during the entire storage period.

### 3.4 Hydrogen peroxide

**Figure 3:**
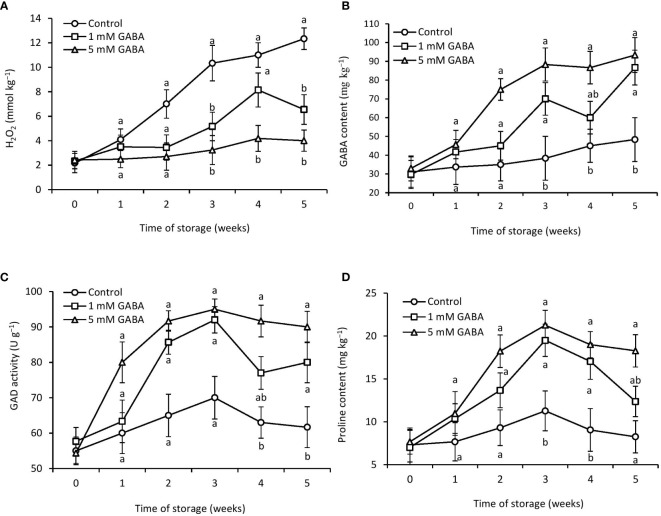
H_2_O_2_ **(A)**, GABA content **(B)**, GAD activity **(D)**, and proline content **(C)** in papaya fruit treated with GABA during storage at 4°C for 5 weeks. Vertical bars represent the standard error of the means for three replicates. Means with different letters show significant differences.

Chilling injury is an oxidative physiological disorder that induces a significant production of ROS, including hydrogen peroxide, and this leads to lipid peroxidation. Excess production of H_2_O_2_ involves substantial damage to cell membrane stability. Lipid peroxidation caused by ROS generation is revealed by the deterioration of the cell membrane. The control fruit’s H_2_O_2_ level was significantly higher than that of the GABA-treated fruit (**Figure 3A**). After being stored for 5 weeks, papaya fruit treated with 1 mM or 5 mM GABA had H_2_O_2_ levels that were 46% and 67% lower, respectively, than those in the control fruit.

### 3.5 GABA content and GAD activity

Throughout the whole storage period, the amount of endogenous GABA in the control and fruit treated with 1 mM or 5 mM GABA gradually increased. After 5 weeks of storage, the GABA-treated fruit had considerably more GABA than the control fruit (**Figure 3B**). The control fruit showed the lowest endogenous GABA content throughout the storage time. In contrast, the endogenous GABA content of papaya fruit treated with 1 mM or 5 mM GABA was 44% and 48% higher, respectively, than that of the control group after 5 weeks of storage. Exogenous application of GABA treatment potentially affected GAD enzyme activity. Initially, the activity of the GAD enzyme increased from the first week to the third week in both treated and untreated fruit and then declined until the end of cold storage (**Figure 3C**). However, for the entire storage period, papaya fruit treated with 1 mM or 5 mM GABA significantly maintained higher GAD activity compared to control fruit.

### 3.6 Proline content

Proline content of treated and untreated fruit increased until the first 3 weeks of storage and then declined. The proline content was 34% and 54% higher in fruit subjected to 1 mM or 5 mM GABA, respectively, than the control after storage for 5 weeks (**Figure 3D**).

### 3.7 Antioxidant enzyme activities

**Figure 4:**
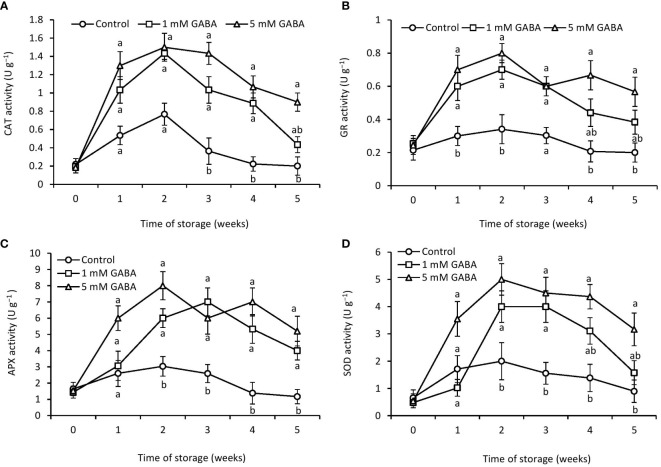
Activities of CAT **(A)**, GR **(B)**, APX **(C)**, and SOD **(D)** in papaya fruit treated with GABA during storage at 4°C for 5 weeks. Vertical bars represent the standard error of the means for three replicates. Means with different letters show significant differences.

Both treated and untreated papaya fruit initially had higher CAT activity during the first 2 weeks, which then steadily decreased until the end of the storage period (**Figure 4A**). However, this declining rate was more obvious in the control fruit. Papaya fruit treated with 1 mM or 5 mM GABA substantially maintains a high level of CAT activity. GR plays a key role against ROS in the defense system and actively takes part in the ascorbate-glutathione (ASH-GSH) cycle. The results showed that GR activity was positively affected by GABA treatment (**Figure 4B**). Firstly, GR activity increased in all papaya fruit, reached its peak after 2 weeks of storage, and then progressively declined during the rest of the storage period. GR activity in papaya fruit treated with 1 mM or 5 mM GABA was 32% and 64% higher, respectively, than the control fruit at 5 weeks of storage period.

In both GABA-treated and control fruit, APX activity increased during the first 2 weeks, then dropped until the end of the storage period (**Figure 4C**). However, this decreasing rate was more prominent in the control fruit. Papaya fruit treated with 1 mM or 5 mM GABA enhanced APX activity more than that in the control fruit. SOD is considered one of the most important defense-related enzymes and crucial for the detoxification of ROS during stress conditions. Exogenous application of GABA treatment (1 mM or 5 mM) maintained a higher level of SOD activity than that in the control fruit over the entire storage period (**Figure 4D**).

### 3.8 PAL and PPO activity

**Figure 5:**
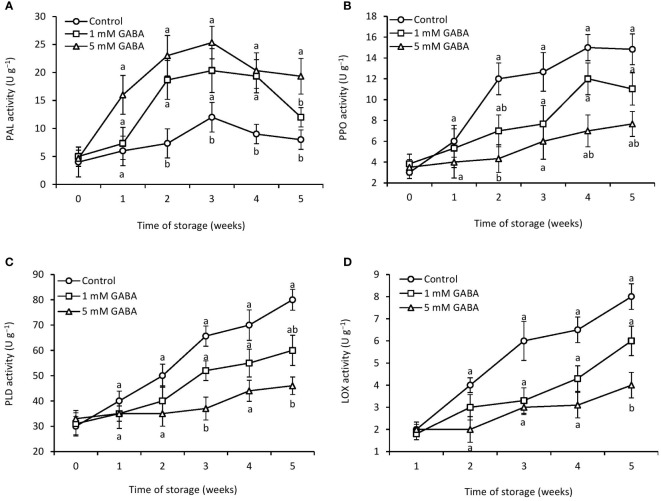
Activities of PAL **(A)**, PPO **(B)**, PLD **(C)**, and LOX **(D)** in papaya fruit treated with GABA during storage at 4°C for 5 weeks. Vertical bars represent the standard error of the means for three replicates. Means with different letters show significant differences.

Up to 3 weeks of storage, PAL activity in the GABA-treated and control fruit increased gradually; thereafter, it dropped until the end of the storage period (**Figure 5A**). Compared with the control fruit, papaya fruit treated with 1 mM or 5 mM GABA triggered PAL activity during the whole storage time. PPO activity gradually increased over the course of 4 weeks in both the GABA-treated and control groups (**Figure 5B**). After that, it slightly declined in control and fruit treated with 1 mM GABA. The PPO activity of papaya fruit treated with 1 mM or 5 mM GABA was 21% and 50% lower, respectively, than that of the control group after 5 weeks of storage, which indicated that GABA could limit PPO activity during storage.

### 3.9 PLD and LOX enzyme activity

PLD activity increased with an increase in the storage period. However, papaya fruit treated with GABA reduced the PLD activity (**Figure 5C**). For the whole storage period, fruit exposed to 5 mM GABA showed the lowest level of PLD activity. After being stored for 5 weeks, papaya fruit treated with 1 mM or 5 mM GABA may have reduced the LOX activity compared to the control fruit (**Figure 5D**), which demonstrates that GABA may be able to restrict LOX activity during storage.

### 3.10 Total phenolics and antioxidant activity

**Figure 6:**
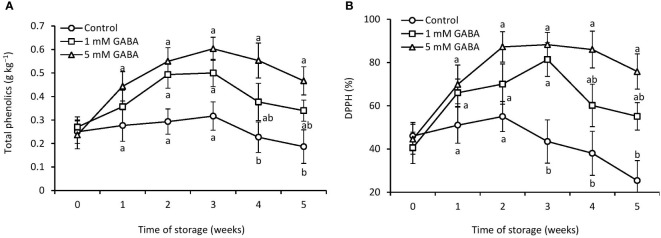
Total phenolics **(A)** and DPPH **(B)** in papaya fruit treated with GABA during storage at 4°C for 5 weeks. Vertical bars represent the standard error of the means for three replicates. Means with different letters show significant differences.

Over the first 3 weeks of storage, phenolic contents steadily increased in all papaya fruit but thereafter decreased (**Figure 6A**). However, over the entire storage period, the GABA treatment (1 mM or 5 mM) preserved higher levels of total phenolic contents than the control fruit. The DPPH-radical scavenging activity of GABA-treated fruit reached its peak after 3 weeks of storage and then steadily decreased (**Figure 6B**). However, the highest DPPH activity peak was observed in the control fruit after 2 weeks of storage. The DPPH activity was 26% and 67% higher in fruit exposed to 1 mM or 5 mM GABA, respectively, than the control after storage for 5 weeks.

## 4 Discussion

The present results indicated that GABA treatments (1 mM or 5 mM) improved the chilling tolerance of papaya fruit during low-temperature storage. The symptoms of chilling injury on the fruit surface and internal browning were clearly inhibited in treated papaya fruit. Similar evidence has been observed in other fruits treated with GABA, such as peach (Shang et al., 2011), banana (Wang et al., 2014), and cucumber (Malekzadeh et al., 2017). The correlation between chilling injury and defense system-related indices of papaya fruit was performed using Pearson correlation analysis. In this study, proline and DPPH were positively correlated with chilling injuries. However, a negative correlation was observed between chilling injury and GABA, GAD, CAT, GR, APX, SOD, and phenolics (**Table 1**). Exogenous application of GABA enhanced tolerance to chilling stress and improved the GABA shunt pathways in many fruits (Ramos-Ruiz et al., 2019). The cell membrane is the first line of defense to protect the cell during stress. The primary event that happens during chilling stress is the destruction of the plasma membrane (Mditshwa et al., 2023). When chilling-sensitive plants are subjected to low-temperature stress, the cell membrane modifies from a flexible structure to a rigid one, and this could cause cracks and leakage of water, ions, and metabolites (Wolfe, 1978). Under cold stress, changes in cell membrane structure have an effect on membrane fluidity and performance (Liang et al., 2020). Electrolyte leakage is used as a parameter for the assessment of cell membrane stability and permeability. Degradation of cell membrane structure occurs by changing the proportion of unsaturated/saturated fatty acids and the increasing level of electrolyte leakage (Marangoni et al., 1996). The involvement of GABA in maintaining cell membrane integrity and fluidity has been widely studied. For example, GABA treatments improved cold tolerance in blood orange and pear fruits by protecting cell membrane structure (Habibi et al., 2019; Li et al., 2019). GABA treatment may inhibit electrolyte leakage and subsequently maintain cell membrane integrity in treated papaya fruit.

**Table 1:** Pearson correlation coefficients between chilling injury and defense system-related indices of papaya fruit during storage.

	GABA	GAD	Proline	CAT	GR	APX	SOD	PAL	Phenolic	DPPH	CI
GABA	–										
GAD	0.48	–									
Proline	0.55	0.68	–								
CAT	0.85	0.54	0.73	–							
GR	0.91	0.61	0.75	0.95	–						
APX	0.92	0.54	0.77	0.73	0.69	–					
SOD	0.39	0.72	0.87	0.89	0.86	0.72	–				
PAL	0.60	0.65	0.89	0.70	0.62	0.69	0.74	–			
Phenolic	0.92	0.82	0.89	0.90	0.91	0.72	0.93	0.67	–		
DPPH	−0.19	−0.08	−0.05	−0.10	−0.09	−0.01	0.06	−0.13	−0.15	–	
CI	−0.41	−0.02	0.01	−0.32	−0.35	−0.08	−0.11	−0.23	−0.28	0.56	–

GABA, γ-aminobutyric acid; GAD, glutamate decarboxylase; CAT, catalase; GR, glutathione reductase; APX, ascorbate peroxidase; SOD, superoxide dismutase; PAL, phenylalanine ammonia lyase; DPPH, 2-diphenyl-1-picrylhydrazyl; CI, chilling injury. p < 0.01.

MDA is a byproduct of lipid peroxidation and a stress marker for oxidative cell damage. Peroxidation of cell membrane lipids is one of the primary biochemical signs of chilling injury, and increased MDA generation is a sign that the cell membrane has been damaged (Khaliq, 2015; Ramos-Ruiz et al., 2019). The degradation of lipids and peroxidation of unsaturated fatty acids are the major reasons for membrane breakdown. MDA is the final product of peroxidation that determines membrane deterioration and oxidative damage in response to stress. The main causes of membrane breakdown are lipid degradation and unsaturated fatty acid peroxidation. MDA is responsible for oxidative damage and membrane degradation in response to stress. Evidence has shown that the increased accumulation of MDA and ion leakage is closely associated with chilling stress in plants (Ding et al., 2007). An increase in ion leakage and MDA has been observed in many fruits and vegetables suffering from chilling injury (Valenzuela et al., 2017). Recent studies have revealed that elicitors like nitric oxide, melatonin, and methyl jasmonate inhibited the occurrence of chilling injury and regulated stress responses in various horticultural crops, including banana (Wang et al., 2016), sapota (Mirshekari et al., 2020), and pomegranate (Chen et al., 2021). The same behavior has been found in cornelian cherry fruit, where GABA treatment postponed MDA accumulation and electrolyte leakage (Rabiei et al., 2019). These results indicated that ion leakage and lipid peroxidation intensified the manifestation of chilling injury in control fruit. However, papaya fruit treated with GABA may lessen membrane lipid breakdown and solute leakage, improving the fruit’s ability to adapt to low-temperature stress.

Both regular cell metabolism and numerous biotic and abiotic stress conditions result in the production of ROS (Hilal et al., 2023). ROS plays a dual role as both beneficial and harmful, depending on their concentration in plant cells. Low concentrations of ROS function as a secondary messenger in signaling transduction pathways that mediate numerous physiological responses in plants (Apel and Hirt, 2004). However, the disproportionate production of ROS during stress can perturb cell homeostasis and even lead to cell death (Aghdam et al., 2016). Therefore, it is necessary to maintain balanced levels of ROS within a certain range to ensure normal cell metabolism. During chilling stress, overproduction of ROS causes oxidative damage to chloroplasts, mitochondria, and apoplast and decreases the activities of antioxidant enzymes, thus destroying the membrane system, causing metabolic disorders, and ultimately leading to cell lysis (Blokhina et al., 2003). Several evidences have shown that chilling injury in fruits can be partly attributed to the imbalance production of ROS during stress. Chilling stress increases ROS levels that stimulate lipid peroxidation and eventually result in the destruction of cell membranes (Mohammadrezakhani et al., 2019). ROS increases the peroxidation of membrane lipids and oxidative damage by producing hydroxyl radicals (Malekzadeh et al., 2017). GABA treatment decreased the accumulation of H_2_O_2_ and enhanced the cold adaptation mechanism in blood orange, pear, and anthurium cut flowers (Habibi et al., 2019; Li et al., 2019; Mahjoory et al., 2019). In the present experiment, the trend of H_2_O_2_ was similar to the results of Rabiei et al. (2019), where cornelian cherry fruit was treated with GABA. These results demonstrated that GABA treatment apparently delayed H_2_O_2_ in treated fruit. Hence, the results suggest that GABA may scavenge H_2_O_2_ and other ROS, resulting in less oxidative damage in treated papaya fruit.

In response to biotic and abiotic stresses, plants’ endogenous GABA content was found to increase. The biosynthetic pathways of GABA were improved through the exogenous GABA shunt by the provision of carbon skeletons and energy (Ramos-Ruiz et al., 2019). GABA plays a crucial part in several pathways by controlling the generation of ROS, increasing energy status, and enhancing the activities of antioxidant enzymes against stresses (Li et al., 2019). The cytosolic enzyme GAD is mainly responsible for the biosynthesis of endogenous GABA (Sheng et al., 2017). Several studies reported that endogenous GABA accumulation increased in fruits treated with GABA, like cucumber and pear (Malekzadeh et al., 2017; Li et al., 2019). Papaya fruit improves low-temperature tolerance by enhancing the activities of energy metabolism-related enzymes like GAD (Pan et al., 2019). The GAD enzyme was increased in peach and banana fruits by improving the endogenous GABA content (Shang et al., 2011; Ali et al., 2022). Aghdam et al. (2016) reported that the GABA shunt pathway might be a potent mechanism for enhancing chilling tolerance in fresh produce. In this study, the exogenous GABA treatment could be one of the reasons that induced the GABA-shunt pathway by stimulating GAD enzyme activity in treated papaya fruit.

Proline is the main amino acid that is responsible for membrane stabilization, free-radical scavenging activity, and cellular osmotic regulation in plants. Proline plays an important role during stress and positively contributes to stress responses, such as chilling injuries. Mohammadrezakhani et al. (2019) reported that exogenous proline treatment reduced the deleterious effects of oxidative stress in citrus fruit by activating antioxidant enzyme activities. Proline has a strong role in the mediation of chilling tolerance by regulating defense responses. In plants, pyrroline-5-carboxylate synthetase and ornithine δ-aminotransferase enzymes are responsible for the biosynthesis of proline. Peach fruit treated with GABA enhanced proline content due to increased activity of pyrroline-5-carboxylate synthetase and ornithine δ-aminotransferase enzymes (Shang et al., 2011). Elevated proline content under the regulation of proline metabolism may be linked with chilling stress resistance, as reported in oxalic acid-treated mango fruit (Li et al., 2014) and litchi fruit (Liu et al., 2020). The effect of GABA on regulating the proline content has been observed in several fruits stored under low-temperature stress. Proline content was improved in GABA-treated papaya. Therefore, treated fruit indicated a higher chilling tolerance, which could be partly connected with the induced proline content. These results agree with those of Shang et al. (2011) and Wang et al. (2014), who reported a higher content of proline in peach and banana fruits treated with GABA.

Plant cells contain an enzymatic defense system against the overproduction of ROS during biotic and abiotic stresses (Blokhina et al., 2003). The antioxidant enzymes, e.g. CAT, APX, GR, and SOD are the primary constituents of the defense system that protect fruits against ROS (Apel and Hirt, 2004). Generally, when fruits are exposed to chilling stress, the activities of antioxidant enzymes, including CAT, GR, APX, and SOD, may be induced. Antioxidant enzymes actively participate in the defense mechanism. GR and APX are the key components of the ascorbate-glutathione cycle and play a crucial role in scavenging ROS (Yang et al., 2011). Catalase is an essential antioxidant enzyme for scavenging ROS during stress. CAT is the main part of a defense system that directly dismutases H_2_O_2_ into water and oxygen (Rabiei et al., 2019). SOD plays a protective role and detoxifies free radicals during stress. SOD catalyzes the superoxide anion to H_2_O_2_, and that H_2_O_2_ can be scavenged by APX or CAT. Generally, under low-temperature stress, the increased activities of antioxidant enzymes are correlated with chilling tolerance. The higher activity of antioxidant enzymes in mango, peach, and papaya fruits was positively related to the acquisition of chilling tolerance (Ding et al., 2007; Yang et al., 2011; Shadmani et al., 2015). It was reported that GABA has an essential role in reducing biotic and abiotic stress, regulating metabolic processes, and increasing chilling tolerance (Aghdam et al., 2016). It was known that endogenous GABA concentrations quickly accumulated in response to chilling stress in peach, citrus, and sapota fruits, and the increased level of GABA was involved in cold adaptation mechanisms (Shang et al., 2011; Mohammadrezakhani et al., 2019; Mirshekari et al., 2020). These findings support the idea of Malekzadeh et al. (2017), who found that cucumbers treated with GABA increased resistance to chilling stress by stimulating the activities of antioxidant enzymes. These results can be clarified by the evidence that GABA treatment could enhance the antioxidant enzyme activities, which in turn could scavenge the excess production of ROS and thus protect the papaya fruit from oxidative damage.

PAL is considered a biochemical marker that induces resistance mechanisms in fruits and vegetables during low-temperature stress. PAL activity is increased in response to low-temperature stress in orange fruit (Habibi et al., 2019). PAL is the major enzyme responsible for the biosynthesis of phenols (Nguyen et al., 2003). PAL activity is positively correlated with the phenylpropanoid pathway (Sun et al., 2020). The phenylpropanoid pathway has been involved in the production of phenols such as lignin, hydroxycinnamic acids, flavonoids, isoflavonoids, and coumarins (Anthony et al., 2023). These phenolic metabolites are implicated in the defense system and antioxidant activity. In this study, the stimulation of PAL activity through the application of GABA treatment could be associated with the increased production of metabolites that would help increase chilling tolerance in papaya fruit. PPO is the main enzyme responsible for the flesh and peel browning of fruits and vegetables. Peeling and flesh browning are the prominent symptoms of chilling injury during storage at low temperatures. The major cause of fruit and vegetable browning is the oxidation of phenol by the PPO (Nguyen et al., 2003). Under stress conditions, the PPO enzyme oxidizes monophenol to diphenol and again oxidizes diphenol to quinones, and these react with proteins or amino acids, which results in brown pigmentation (Valenzuela et al., 2017). The increased activity of PPO is closely linked to the advanced chilling injury symptoms in pomegranate stored at low-temperature stress (Chen et al., 2021). It has been reported that exogenous GABA treatments reduced the PPO activity in blood orange (Habibi et al., 2019). The reduced activity of PPO in papaya fruit treated with GABA indicates that it is connected to a low incidence of chilling injury.

During senescence and stress, the activity of PLD increases, which causes membrane breakdown. PLD initiates the hydrolysis of lipids under salt and chilling stresses (Mirshekari et al., 2020). A lipolytic cascade causes membrane damage as PLD and LOX are critical for the breakdown of phospholipids (Bargmann et al., 2009). LOX plays an essential role in the oxidative breakdown of membrane lipids. LOX activity enhances lipid unsaturation and membrane fluidity (Malekzadeh et al., 2017). The stimulation of PLD and LOX results in irreparable membrane destruction and eventually the manifestation of chilling injury (Aghdam et al., 2016). These findings imply that PLD and LOX may contribute to the development of chilling symptoms in papaya fruit. GABA treatments improve chilling tolerance in papaya fruit by reducing the activity of PLD and LOX enzymes. Thus, it can be inferred that GABA treatment may be an innovative approach to reducing papaya fruit oxidative damage during low-temperature storage due to its reducing effects on PLD and LOX enzyme activity.

Phenolic contents have antioxidant properties and scavenge reactive oxygen species. Phenolic compounds retain the nutritive qualities of fruits and vegetables, for example, flavor, color, bitterness, and astringency (Rastegar et al., 2020). Phenolic compounds present in fruits reveal their physiological role and involvement in antioxidant capacity. The Folin–Ciocalteu method is commonly used for the quantitative investigation of total phenolic content in fruits and vegetables. Phenolic contents in untreated fruit first increased and then decreased with the storage period, although GABA treatments induced the increase in phenolic content. A number of studies showed that postharvest treatments reduced the loss of phenolic content and stimulated antioxidant activity in various fruits during cold storage (Hanif et al., 2020; Khaliq et al., 2021). It was reported that GABA treatment detoxified ROS, triggered DPPH-radical scavenging capacity, and retained the phenolic content of banana fruit (Wang et al., 2014). Similarly, GABA treatment reduced the loss of phenolic content and enhanced DPPH-radical scavenging activity in mango fruit (Rastegar et al., 2020). Phenolic content and DPPH-radical scavenging activity first increased and then declined in papaya fruit during storage (Mendy et al., 2019). There are different kinds of nonenzymatic antioxidant compounds that could contribute to the total antioxidant capacity. However, it is not clear which constituents are mainly responsible for increasing antioxidant activity. In this work, the chilling injury was accompanied by higher flesh browning in the control fruit, which might have happened due to phenol oxidation. GABA treatments can postpone the oxidation of phenolics by preventing the commencement of oxidizing chain reactions. The increased accumulation of phenolics in GABA-treated papaya fruit may enhance antioxidant capacity, consequently inhibiting ROS accumulation and improving chilling tolerance.

## 5 Conclusions

The present results demonstrated that GABA treatment played a vital role in improving the chilling tolerance of papaya fruit by inducing a defense system and reducing oxidative damage. The exogenous application of GABA treatment reduced lipid peroxidation, ion leakage, H_2_O_2_, PPO, PLD, and LOX activity. Additionally, the accumulation of proline, endogenous GABA, and total phenolics may be beneficial for maintaining plasma membrane fluidity and integrity. These findings help us better understand the involvement of GABA in the chilling tolerance of papaya stored at low-temperature stress. Therefore, the exogenous GABA treatment is a nontoxic technique to maintain the nutritional value and quality of papaya fruit.

## Data availability statement

The raw data supporting the conclusions of this article will be made available by the authors, without undue reservation.

## Author contributions

GK and SA: conceptualization and writing—original draft. SE and GA: visualization and writing. YF and JM: formal analysis and data curation. MS and AA: reviewing and editing. All authors contributed to the article and approved the submitted version.
